# Increasing socioeconomic inequalities in first acute myocardial infarction in Scotland, 1990–92 and 2000–02

**DOI:** 10.1186/1471-2458-9-134

**Published:** 2009-05-11

**Authors:** Carolyn A Davies, Ruth Dundas, Alastair H Leyland

**Affiliations:** 1 MRC Social and Public Health Sciences Unit, 4 Lilybank Gardens, Glasgow, UK, G12 8RZ

## Abstract

**Background:**

Despite substantial declines, Ischaemic Heart Disease (IHD) remains the largest cause of death in Scotland and mortality rates are among the worst in Europe. There is evidence of strong, persisting regional and socioeconomic inequalities in IHD mortality, with the majority of such deaths being due to Acute Myocardial Infarction (AMI). We examine the changes in socioeconomic and geographic inequalities in first AMI events in Scotland and their interactions with age and gender.

**Methods:**

We used linked hospital discharge and death records covering the Scottish Population (5.1 million). Risk ratios (RR) of AMI incidence by area deprivation and age for men and women were estimated using multilevel Poisson modelling. Directly standardised rates were presented within these stratifications.

**Results:**

During 1990–92 74,213 people had a first AMI event and 56,995 in 2000–02. Adjusting for area deprivation accounted for 59% of the geographic variability in AMI incidence rates in 1990–92 and 33% in 2000–02. Geographic inequalities in male incidence reduced; RR for smaller areas (comparing area on 97.5th centile to 2.5th) reduced from 1.42 to 1.19. This was not true for women; RR increased from 1.45 to 1.59. The socioeconomic gradient in AMI incidence increased over time (p-value < 0.001) but this varied by age and gender. The gradient across deprivation categories for male incidence in 1990–92 was most pronounced at younger ages; RR of AMI in the most deprived areas compared to the least was 2.6 (95% CI: 1.6–4.3) for those aged 45–59 years and 1.6 (1.1–2.5) at 60–74 years. This association was also evident in women with even stronger socioeconomic gradients; RRs for these age groups were 4.4 (3.4–5.5), and 1.9 (1.7–2.2). Inequalities increased by 2000–02 for both sexes; RR for men aged 45–59 years was 3.3 (3.0–3.6) and for women was 5.6 (4.1–7.7)

**Conclusion:**

Relative socioeconomic inequalities in AMI incidence have increased and gradients are steepest in young women. The geographical patterning of AMI incidence cannot be fully explained by socioeconomic deprivation. The reduction of inequalities in AMI incidence is key to reducing overall inequalities in mortality and must be a priority if Scotland is to achieve its health potential.

## Background

Ischeamic heart disease (IHD) mortality trends have fallen in developed countries over recent decades; this is mainly due to a decrease in disease incidence through improved primary prevention measures and to a drop in case fatality reflecting advances in diagnosis, treatment and secondary prevention[1]. Although downward trends in IHD mortality have been seen in Scotland [2] these have been to a lesser extent than in other Western European countries, with the result that Scotland has one of the worst IHD mortality rates in the region[3]. Scotland was found to have among the highest rates of fatal and non-fatal events when comparing the MONICA populations [4]. However, survival rates from IHD in Scotland make for a more favourable comparison; results from the MONICA study showed that 28-day case fatality in Scotland was the same as or lower than the average across all populations. This suggests that high incidence has been driving Scotland's high IHD mortality.

Incidence rates of acute myocardial infarction (AMI), the most common form of IHD, have also declined substantially over recent years[2]. This mainly reflects improvements in primary prevention of the disease; for example, there have been intensive lifestyle interventions to reduce levels of exposure to risk factors such as smoking[5]. To bring Scotland's IHD mortality rates to a rate comparable with other European countries the downward trends in first time AMI events must continue, ideally at a faster pace.

Given the Scottish Government's commitment to tackling inequalities in health[6] and the contribution inequalities make to overall mortality[7], it is important to explore whether similar declines in AMI incidence are being experienced by all population groups within the country. Incidence is an important health indicator when examining the burden and patterning of a disease; however, to date, studies in Scotland have mainly focused on various inequalities in cardiovascular death [8-10] or survival[8,11-13] and treatment[14] of AMI or IHD. Little is known about social and geographical inequalities in AMI incidence and how they interact with age, gender and time. If such inequalities in first time AMI events have increased in Scotland it would suggest that improvements in primary prevention of the disease have not been experienced equally by all sections of Scottish society.

Population based studies of AMI incidence are uncommon but Scotland is in the advantageous position of having a data system permitting the linkage of information on hospital admissions with mortality data for the whole population (5.1 million in 2001[2]). The aim of this work is to use these data to examine the changes in socioeconomic and geographic inequalities in incidence of AMI in Scotland between 1990–92 and 2000–02 and to explore how these inequalities interact with age and gender.

## Methods

### Data

The data were obtained from the Scottish system of linked hospital discharge records. These were provided by the Information Services Division, NHS National Services Scotland, following successful application to their Privacy Advisory Committee. The Information and Services Division routinely links multiple hospital discharges for the same individual and links hospital discharges to mortality data provided by the General Register Office for Scotland[15]. Such data permit the identification of patient careers covering more than one spell of hospital care. We used these data to identify all IHD events – hospital discharges or deaths – in Scotland between 1981 and 2004. Our interest was to examine the patterning of AMI incidence, this being the main form of IHD. We defined AMI incidence as a first time attack within a 7 year period with AMI (ICD9: 410, ICD10: I21–I22) as the primary or secondary diagnosis at discharge or as the underlying or contributory cause of death, or with other IHD (ICD9:410–414; ICD10: I20–I25) as the underlying cause of death[16]. This definition of AMI incidence includes both AMI events and IHD deaths; on the death certificate AMI deaths may be coded as other forms of IHD therefore the inclusion of this group means we are less likely to miss any AMI deaths which were incident events. Our data provide information on 1,035,692 IHD events between 1981 and 2004. The linkage from 1981 enabled us to identify retrospectively individuals aged over 29 years who had had an incident AMI event (i.e. for whom there was no hospital admission with a primary diagnosis of AMI in the previous 7 years) between 1988 and 2004, giving a total of 376,040 incident events. For each individual with an event we had information on age, sex, postcode and Local Council Area (LCA) of residence, and on dates of admission and discharge (for patients admitted to hospital) and death if it occurred.

Postcode sectors (mean population 5402, range 53–20,512) were used to allocate individuals into 7 deprivation categories (DEPCATs) using Carstairs scores of socioeconomic deprivation[17,18]. These scores were derived from measures of overcrowding, male unemployment, households without a car and low social class at each Census (1991 and 2001). Details of the construction of the score can be found elsewhere[19]. At each Census, approximately 6% of the Scottish population lived in areas described as DEPCAT 1 (the least deprived) and 7% in the most deprived (DEPCAT 7). Since we were dependent on deprivation scores derived from the Censuses we restricted our comparisons to those years around the Census, 1990–92 and 2000–02. We had information on 74,213 AMI incidents in people aged over 29 years in 1990–92 and 56,995 in 2000–02. In 1990–92, 873 patient records (1.2%) had missing postcode information and were excluded from our analysis; the corresponding figure for 2000–02 was 437 (0.8%).

### Statistical Analysis

We analysed the data using multilevel modelling in MLwiN [20] to take account of the hierarchical data structure. Incidence was modelled within a hierarchy of age and gender groups (a pseudolevel) nested within postcode sectors within LCAs; the rates for postcode sectors within the same LCA are likely to be correlated, as are the rates for age and gender groups within a given postcode sector. Geographic inequalities were assessed through the partitioning of the variation in incidence rates to that attributable to each of postcode sector (n = 1010) and LCA (n = 32) levels. A larger variance at a given level is associated with greater geographical inequalities. Risk ratios comparing a notional geographic unit lying on the 97.5th centile with that of an area lying on the 2.5th centile were also used to quantify the geographic variation. Incidence was modelled using Poisson multilevel regression[21] and we adjusted for age, gender and deprivation category (DEPCAT 1-least: DEPCAT 7-most) and first and second order interactions between these. Risk ratios from these models were used to assess the relative socioeconomic inequalities by age and gender. The ratios compare rates in each DEPCAT to DEPCAT 1 (the least deprived areas). Relative inequalities are often affected by the size of the underlying rates therefore we also present directly standardised rates. These are shown for each age, gender and DEPCAT stratification allowing exploration of absolute inequalities (the difference between rates in the most and least deprived areas).

## Results

### Differences by age, sex, deprivation and time

Table 1 shows the total population, the number of incident events and age standardised incidence rates for each year group stratified by sex, age and deprivation group. In 1990–92 the rate was 690 per 100,000; this decreased by 30% to 481 per 100,000 by 2000–02. There was an association between incidence rates and each of sex, age and deprivation at both time points as shown by the *p*-values obtained from univariable models. Risk ratios (RRs) and 95% confidence intervals are also presented for each factor and all differ significantly from one. For example, in 2000–02, women were 31% less likely to have a first AMI than men (RR = 0.69; 95% C.I. 0.67–0.70), those aged 45–59 years old were six times as likely to have a first AMI as people aged 30–44 years (RR = 6.01; 95% C.I. 5.70–6.34) and those living in the most deprived areas were 94% more likely to have a first AMI than those in the least deprived areas (RR = 1.94; 95% C.I. 1.76–2.15).

**Table 1:** Baseline characteristics of population and AMI incidence in 1990–92 and 2000–02 and univariable model results

	Population (%)	Incidence (%)	Std Rate^†^	*p*-value	RR	95% CI
1990–92						
All incidents	8921481	74213	690			
Gender				<0.0001		
Men (ref)	4141974 (46)	41151 (55)	970		1.00	
Women	4779507 (54)	33062 (45)	474		0.69	0.68, 0.70
Age (years)				<0.0001		
30–44 (ref)	3263541 (37)	1874 (3)	59		1.00	
45–59	2557551 (29)	11384 (15)	441		7.73	7.36, 8.12
60–74	2117613 (24)	29660 (40)	1370		24.3	23.2, 25.4
75+	982776 (11)	31295 (42)	3213		55.7	53.1, 58.3
Deprivation				<0.0001		
1 – least (ref)	565935 (6)	3294 (4)	513		1.00	
2	1264680 (14)	8917 (12)	572		1.21	1.12, 1.31
3	1992501 (22)	15754 (21)	635		1.36	1.26, 1.46
4	2264034 (25)	19087 (26)	696		1.47	1.37, 1.59
5	1304034 (15)	12101 (16)	769		1.60	1.48, 1.73
6	978408 (11)	9461 (13)	807		1.63	1.50, 1.78
7 – most	551889 (6)	5599 (8)	881		1.74	1.58, 1.92

2000–02						
All incidents	9618498	56995	481			
Gender				<0.0001		
Men (ref)	4486074 (47)	31847 (56)	673		1.00	
Women	5132424 (53)	25148 (44)	325		0.69	0.67, 0.70
Age (years)				<0.0001		
30–44 (ref)	3488874 (36)	1616 (3)	47		1.00	
45–59	2929725 (30)	8079 (14)	276		6.01	5.70, 6.34
60–74	2123298 (22)	19574 (34)	891		20.0	19.9, 21.0
75+	1076601 (11)	27726 (49)	2550		56.3	53.5, 59.2
Deprivation				<0.0001		
1 – least (ref)	612279 (6)	2570 (5)	335		1.00	
2	1372263 (14)	6504 (11)	370		1.14	1.05, 1.23
3	2154495 (22)	11927 (21)	435		1.35	1.26, 1.46
4	2423592 (25)	14393 (25)	487		1.46	1.35, 1.57
5	1387467 (14)	9404 (16)	554		1.64	1.51, 1.77
6	1063713 (11)	7548 (13)	579		1.72	1.58, 1.87
7 – most	604689 (6)	4649 (8)	674		1.94	1.76, 2.15

^† ^Age standardised rates (per 100,000)

The tables and figures presented in this paper show results stratified by year (1990–90 and 2000–02); however, to explore the interaction between year and deprivation we modelled the unstratified data. This interaction coefficient was significant (*p-*value < 0.001) and positive which suggests that the socioeconomic gradient in AMI incidence increased over time.

### Geographic variations

Table 2 presents the results from fitting three multilevel models for 1990–92 and 2000–02 separately. The full model includes the fixed effects of sex, deprivation category and age group, and each first and second order interaction. For 1990–92, the *p*-value for the interaction between the three fixed effects was highly significant (*p *< 0.001) suggesting that the deprivation inequalities in AMI incidence differed between age groups and that this relationship differed for men and women. Separate models were fitted for each sex and within each model the interaction between DEPCAT and age group was highly significant (*p *< 0.001). A similar picture was evident for 2000–02. Exploring the random effects showed that for men in 1990–92 around half (51%) of the area variation in AMI incidence rates in Scotland was due to differences between the larger geographical areas (LCAs). By 2000–02 this proportion had increased slightly to 55%. For women, there was less regional patterning in 1990–92 (31% of the area variation was between LCAs) and, as for men, this partitioning only changed slightly (to 28%) by 2000–02.

**Table 2:** Poisson multilevel model results exploring socioeconomic interactions with age and gender, and geographic variations in AMI incidence rates

	1990–92				2000–02			
Model	Estimate	*p*-value	ICC^†^	RR^‡^	Estimate	*p*-value	ICC^†^	RR^‡^
Full								
*Fixed*								
Gender		<0.001				0.001		
DEPCAT		<0.001				<0.001		
Age		<0.001				<0.001		
Gender*DEPCAT		<0.001				0.027		
Gender*Age		<0.001				<0.001		
DEPCAT*Age		<0.001				<0.001		
Gender*DEPCAT*Age		<0.001				0.005		
								
*Random*								
Council Area	0.003	0.003	0.29	1.24	0.002	0.001	0.38	1.19
Postcode Sector	0.007	<0.001	0.71	1.39	0.001	<0.001	0.62	1.13
Age/Sex group	1				1			

Men								
*Fixed*								
DEPCAT		<0.001				<0.001		
Age		<0.001				<0.001		
DEPCAT*Age		<0.001				<0.001		
								
*Random*								
Council Area	0.008	0.001	0.51	1.42	0.002	0.002	0.55	1.19
Postcode Sector	0.008	<0.001	0.49	1.42	0.002	<0.001	0.45	1.19
Age/Sex group	1				1			

Women								
*Fixed*								
DEPCAT		<0.0001				<0.001		
Age		<0.0001				<0.001		
DEPCAT*Age		<0.0001				<0.001		
								
*Random*								
Council Area	0.004	0.007	0.31	1.28	0.003	0.005	0.28	1.23
Postcode Sector	0.009	<0.001	0.69	1.45	0.014	<0.001	0.72	1.59
Age/Sex group	1				1			

^† ^The Intraclass Correlation Coefficient (ICC) is the percentage of unexplained residual variance attributable to each higher level in the model.

^‡ ^The Risk Ratio (RR) given at the higher level of local council area (LCA) compares the risk of incidence in a notional LCA lying on the 97.5^th ^centile with a LCA on the 2.5^th ^centile. The RR at the postcode sector (PS) level compares the risk of incidence in a notional PS (within a given LCA) lying on the 97.5^th ^centile with a PS (within the same LCA) on the 2.5^th ^centile.

Note that coefficient estimates are not given for the fixed effects as these consist of numerous dummy variables.

An alternative, more meaningful, approach to quantifying the variance terms at the higher geographical levels in the model is to calculate a risk ratio which compares a notional geographic unit lying on the 97.5th centile, say, with that of an area on the 2.5th centile. These RRs are given in table 2. For example, in 1990–92, the risk of AMI incidence among men within a postcode sector lying on the 97.5th centile in a given LCA is 42% higher than a postcode sector lying on the 2.5th centile. By 2000–02 the risk ratio had fallen to 1.19. Similar geographic variation was evident when comparing larger areas (LCAs) with extreme AMI incidence rates. In contrast, the RR comparing extreme rates of AMI incidence in women between postcode sectors increased from 1.45 in 1990–92 to 1.59 in 2000–02. There was also a smaller reduction in geographic inequalities at the LCA level than was evident for men (1.28 in 1990–92 and 1.23 in 2000–02).

For each time period models with only age and sex and models with age, sex and DEPCAT were also fitted; the estimates from these showed that adjusting for DEPCAT accounted for 59% of the geographic variation in AMI incidence in Scotland in 1990–92 and 33% in 2000–02.

### Socioeconomic inequalities

**Figure 1:**
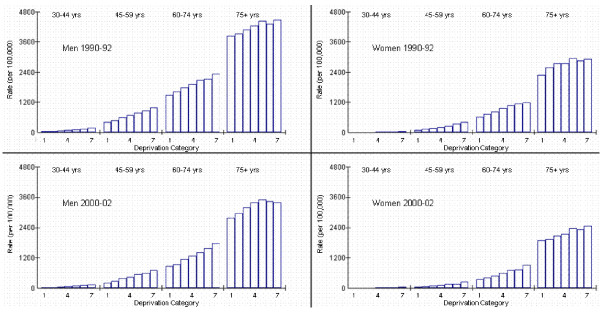
**Age standardised AMI incidence rates by year, gender, age and deprivation**. Rates are directly age standardised to the European standard population and presented per 100,000 population.

**Figure 2:**
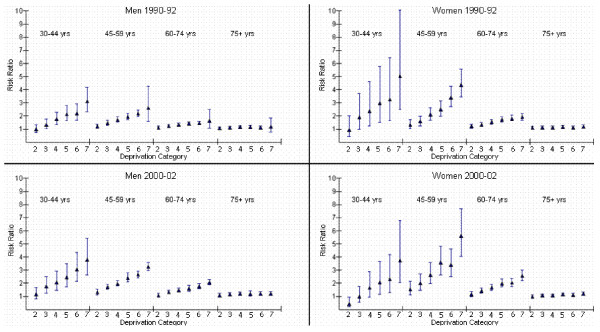
**Socioeconomic gradients in AMI incidence rates by year, gender and age**. Risk ratios and their 95% confidence intervals compare AMI incidence rates in each DEPCAT to DEPCAT 1 (reference category). DEPCAT 1 are the least deprived areas, DEPCAT 7 the most deprived.

In figures 1 and 2 we explore the interaction terms present in the four models split by year and sex; 1990–92 Men, 2000–02 Men, 1990–92 Women and 2000–02 Women. Age standardised rates are presented for each age group and DEPCAT in figure 1. Risk ratios (reference category DEPCAT 1) and their 95% confidence intervals are also plotted for each age group and DEPCAT (figure 2). Within each age group and DEPCAT the risk of AMI incidence is compared to the risk in the least deprived areas (DEPCAT 1). In 1990–92, AMI incidence rates for men were significantly higher in the most deprived areas compared to the least deprived areas for each age group except the oldest. The highest relative inequalities were in the youngest age group where rates in DEPCAT 7 were 3.1 times those in DEPCAT 1. These inequalities declined as the population ages. For women during this time period, a similar picture was apparent. These inequalities were steeper for women; AMI incidence rates for women aged 30–44 years in DEPCAT 7 were 5.0 times those in DEPCAT 1 with the corresponding figure for women aged 45–59 years being 4.4 (compared to 2.6 for men).

### Trends in socioeconomic inequalities

Socioeconomic inequalities in AMI incidence in men increased in each age group between 1990–92 and 2000–02. Rates in DEPCAT 7 in 2000–02 rose to 3.8 times those in DEPCAT 1 at ages 30–44 and 3.3 at ages 45–59. The socioeconomic inequalities in AMI incidence in women aged 45–59 and 60–74 years in 2000–02 were again steeper than for men. There was also an increase in inequalities over time for women aged 45–59 and 60–74 years, with RRs increasing to 5.6 and 2.6 respectively.

Relative inequalities were greatest at younger ages for both sexes and there was a suggestion that these increased over time. Unsurprisingly, absolute inequalities (figure 1) were greater in the older ages where there were many more events. For men in 1990–92, the differences between the age standardised rates in the most and the least deprived areas were 129, 563, 873 and 629 events for 30–44, 45–59, 60–74 and 75+ years respectively. Decreases were not evident in all age groups by 2000–02; the differences in the respective age categories were 104, 492, 934 and 614. The decreases in absolute differences were small considering the large overall drop in AMI incidence during this period. For women in 1990–92 the absolute differences between the most and least deprived areas were 38, 314, 573 and 615, respectively. Again, there were modest decreases in the differences by 2000–02; the corresponding figures were 37, 215, 558 and 567.

## Discussion

This is the largest population based study to explore trends and inequalities in AMI incidence and their interaction with age and gender. Other studies have explored the associations between deprivation and IHD incidence [22-25], the largest of which examined 52,360 individuals who developed IHD (from a study of 2.6 million men and women)[24]. These studies found socioeconomic gradients in incidence but did not report interactions with age and gender or unexplained geographical variation. Within Scotland, most work investigating IHD or AMI to date has focused mainly on mortality [8-10] and case fatality or survival patterns[8,9,11,26].

We have shown that AMI incidence rates have decreased substantially over recent decades; such decreases are likely to be due to improvements in primary prevention of the disease[27,28]. Male incidence rates for those aged 30–44 years old have decreased by 23%, the corresponding rates for those ages 45–59, 60–74 and 75+ years were 37%, 39% and 28%. The figures for women were 5%, 45%, 40% and 25%. IHD mortality also fell more sharply for women aged 45–59 during this time period [10]; mortality rates for men decreased by 44% whilst the rate for women fell by 51%. At older ages the declines were comparable: 44% and 46% respectively for men and women aged 60–74 and 27% and 25% at ages 75+. Although comparing two slightly different disease groups – AMI incidence with IHD mortality – there is a suggestion that there has been a steeper downward trend from 1990–92 to 2000–02 in mortality than incidence. This could partly be due to the increasing use of more sensitive enzyme diagnostic testing, in particular troponin, from the beginning of the current decade resulting in higher hospitalisation rates[29]. More up-to-date data are needed to explore the true effect of troponin on AMI incidence patterning. Regardless of this, reducing the incidence of AMI will have a substantial impact on mortality and, consequently, help reduce the rates in Scotland to a level that is comparable with the rest of Europe.

We have shown that socioeconomic deprivation has a marked effect on the risk of having a first AMI. Relative inequalities across Scotland are steepest in the youngest age groups and most pronounced in young women. Other work looking at IHD mortality has shown that, despite large reductions in rates, there remain strong socioeconomic variations in the disease[30,31]. The steepest inequalities were again found in the 45–59 year old age group in 2000–02 but, contrary to our findings for AMI incidence, these inequalities were higher in men than women[31]. Studies which have examined short-term AMI case fatality have frequently shown stronger inequalities in the young and in particular women[13,32-35].

During the 10-year period under investigation in this paper, inequalities increased with the steepest changes being seen in the younger age groups (under 60). Increasing inequalities are likely to be due to higher levels of exposure of young people in deprived areas to risk factors such as smoking. In Scotland, in both men and women, cigarette smoking prevalence is highest in the 25–34 age group (39% of men and 35% of women), followed by those aged 35–44 years (34% of men and 33% of women)[36]. Between 1995 and 2002 there was a small decrease in the proportion of men aged 16–64 years who smoked (34% to 31%) [37-39] with a comparable decrease for women (36% to 32%). The number of cigarettes smoked per day is significantly associated with deprivation for both sexes with a steeper gradient for women than for men[36], echoing the association between AMI incidence and area deprivation. In 2001, 70% of smokers were in lower socioeconomic groups[37]. Capewell et al[40] quantified the extent to which the fall in IHD mortality in Scotland was attributable to risk factor changes (and how much to medical and surgical treatment). They suggested that 51% of the reduction in mortality over a 20 year period was due to risk factor modifications. This reduction in mortality will largely be due to a reduction in incident events of the disease. They further estimated that, of this 51% reduction, smoking accounted for 36%, a secular fall in blood pressure and cholesterol reduction each contributed 6% and 3% was due to deprivation (susceptible to confounding by smoking, diet and blood pressure changes). Therefore trends and inequalities in smoking habits are likely to explain a large part of the changes in AMI incidence in Scotland over time.

Other traditional risk factors such as diet, physical inactivity, overweight, hypertension or cholesterol [5] may also contribute to the changing patterns of AMI incidence, but to a lesser extent than smoking. For example, there have been substantial increases in obesity, which is associated with diet and physical inactivity, in more deprived areas in Scotland over recent years, with higher rates in women[36]. Also, diabetes rates, which are associated with obesity levels, have increased in Scotland and are associated with deprivation with a suggestion of a stronger gradient in women[36]. Findings from an English cohort study [41], which examined whether deprivation status had an influence on changes in cardiovascular risk factors in middle-aged (35–55 years) men and women, suggested that there had been a widening deprivation gap in populations with high blood pressure. If the same were true in Scotland this could also contribute to the increasing inequalities in AMI incidence. That study also found no association between deprivation and change in cholesterol, and, perhaps somewhat surprisingly, a narrowing of inequalities in smoking in women. Other research has concluded that there is a narrowing of socioeconomic inequalities in high blood pressure rates in men, and in high body mass index and cholesterol in both sexes and a widening of inequalities in high blood pressure in women and smoking in both sexes [42]. All such findings suggest that the relationship between cardiovascular risk factors and deprivation is complex and varies across sex, age and geographical groups. Further work within Scotland examining the relationship between such risk factors and AMI incidence will be important in developing our understanding of inequalities.

Another factor contributing to the inequalities in AMI incidence might be variations in follow-up contact rates with general practitioners (GP) following a diagnosis of angina. It is well known that patients often undergo episodes of unstable or stable angina prior to their first AMI[43] and that young deprived patients (and in particular women) with angina have less frequent follow-up contacts with their GP. For example, one year GP contact rates in Scotland in 2001–02 for angina were higher in men than women, were extremely low in patients under 45 years and declined significantly with increasing socioeconomic deprivation[44]; these factors may all contribute to the inequalities reported here. Although reducing exposure to risk factors is an important primary prevention strategy in all population groups, greater inequalities in AMI incidence at younger ages, and in particular among younger women, would suggest particular emphasis should be made on these sub-groups.

Much, but not all, of the geographic variation in AMI incidence in Scotland can be explained by a simple measure of area socioeconomic deprivation. After accounting for age, sex and deprivation a substantial proportion of the remaining variation in 2000–02 was attributable to differences between LCAs, particularly in women, suggesting strong regional patterning of AMI incidence and warranting investigation into the likely mechanisms linking area of residence to occurrence of first AMI events.

It may be misleading to present relative inequalities alone as these are related to underlying rates and the scale on which the inequalities are measured[45,46]. For example, the number of incident AMI events is much higher in older than younger age groups; therefore, risk ratios (comparing affluent to deprived areas) that are as high as in the younger age groups could only be achieved at older ages if the absolute differences between affluent and deprived areas were considerably larger. We have presented age standardised rates alongside risk ratio comparisons so that absolute differences can also be considered. Absolute inequalities (comparing the most deprived to the most affluent areas) were highest for men aged 60–74 years and have increased despite the decline in incidence rates at these ages between 1990–92 and 2000–02. There were modest decreases in inequalities in all other age groups. Absolute inequalities were highest for women aged 75+ years and the decreases over time in all age groups were small. We feel relative inequalities are more informative here, especially when examining the younger age groups; an absolute difference in AMI incidence at younger ages, which may seem small in comparison to the older age groups, may be of greater public health importance. IHD is a highly preventable disease in the young, so reducing the rates in the more deprived areas to a level similar to that in the more affluent areas should be achievable and is of primary importance.

### Study limitations

One limitation of our study is that we only had an area based measure of deprivation as opposed to individual socioeconomic status. The Carstairs deprivation index is a commonly used measure and has been validated against individual socioeconomic status [47]; however, the population size of the geographic area (postcode sector) for which our deprivation index is derived may be influencing our estimation of the socioeconomic gradient. Estimates of inequalities based on area deprivation have been shown to be diluted when the geographical units are large[48]. The smaller areas in this study, postcode sectors, are still fairly large (mean population 5402) and therefore are likely to be heterogenous. However, the interpretation to be drawn from the analysis does not relate to individual socioeconomic status but to the area context. Previous work has shown the prevalence of cardiovascular disease in Scotland to be related to area deprivation (at the postcode sector level, based on the Carstairs score) and not individual occupational social class [49]. It is unfortunate that the routine data used in this study do not permit adjustment for both individual and contextual measures. Secondly, on admission to hospital, MI is normally diagnosed as non-ST-elevated (non-STEMI) or ST-elevated (STEMI) and the treatment and prognosis differ according to this diagnosis [50-52]. A further important avenue of research would therefore be to investigate how the trends in incidence of STEMI and non-STEMI contribute to the overall trends in MI incidence. Again, it is unfortunate that our routine data do not enable a distinction between STEMI and non-STEMI, and we therefore cannot comment on whether trends and inequalities reflect changes in one or the other. Moreover, we do not have routinely collected data on IHD risk factors for this population and therefore can only hypothesise as to why rates are decreasing and inequalities persisting or increasing. We can also only hypothesise about the contribution that reductions and inequalities in AMI incidence are having on the trends and patterns of AMI mortality in Scotland; further work incorporating case fatality from the disease is needed to answer such questions.

## Conlusion

IHD mortality has fallen in Scotland as in much of the developed world [1]. However, the decreases have not been experienced by all social groups; whilst the overall mortality rate due to IHD fell by 61% among men under 65 between 1980–82 and 2000–02, and by 62% among women of the same ages, rates for both in the most deprived areas fell by just 37% [10]. Both the declines in rates and the relative increases in inequality seem to be driven by changes in incidence of AMI. Reducing AMI incidence among the most disadvantaged populations is therefore key to reducing overall inequalities in mortality and to ensuring that the Scottish population can achieve its health potential.

## Competing interests

The authors declare that they have no competing interests.

## Authors' contributions

CD is the corresponding author and guarantor of this paper. CD formulated the research question, analysed and interpreted the data and wrote the paper. AL initiated the study and commented on the paper. RD helped with data analysis and commented on the paper.

## Pre-publication history

The pre-publication history for this paper can be accessed here:


